# Prevalence and Risk Factors for Colonization by Multidrug-Resistant Microorganisms among Long-Term Travelers and Recently Arrived Migrants

**DOI:** 10.3390/microorganisms12050936

**Published:** 2024-05-04

**Authors:** Víctor Monsálvez, Paula Bierge, María Luisa Machado, Oscar Q. Pich, Elisa Nuez-Zaragoza, Carme Roca, Ana I. Jiménez-Lozano, Ángela Martínez-Perez, Aina Gomila-Grange, Isabel Vera-Garcia, Ana Requena-Méndez, Silvia Capilla, Oriol Gasch

**Affiliations:** 1 Infectious Diseases Department, Parc Taulí Hospital Universitari, Institut d’Investigació i Innovació Parc Taulí (I3PT-CERCA), Universitat Autònoma de Barcelona, 08208 Sabadell, Spain; vmonsalvez@tauli.cat (V.M.); mmachado@tauli.cat (M.L.M.); agomila@tauli.cat (A.G.-G.); 2 Laboratori de Recerca en Microbiologia i Malalties Infeccioses, Parc Taulí Hospital Universitari, Institut d’Investigació i Innovació Parc Taulí (I3PT-CERCA), Universitat Autònoma de Barcelona, 08208 Sabadell, Spain; pbierge@tauli.cat (P.B.); oquijada@tauli.cat (O.Q.P.); 3 Institut de Biotecnologia i Biomedicina, Universitat Autònoma de Barcelona, 08193 Bellaterra, Spain; 4 Microbiology Unit, Parc Taulí Hospital Universitari, Institut d’Investigació i Innovació Parc Taulí (I3PT), Universitat Autònoma de Barcelona, 08208 Sabadell, Spain; enuez@tauli.cat (E.N.-Z.); scapilla@tauli.cat (S.C.); 5 Centre d’Atenció Primaria El Clot, Institut Català de la Salut (ICS), Carrer Concilio de Trento 25, 08018 Barcelona, Spain; croca.bcn.ics@gencat.cat; 6 Facultat de Medicina i Ciències de la Salut, Universitat de Barcelona (UB), Carrer Casanova, 143, 08036 Barcelona, Spain; 7 Centre d’Atenció Primaria Roger, Badal 3I Institut Catala de la Salut ICS Barcelona, 08028 Barcelona, Spain; aijimenez.bcn.ics@gencat.cat; 8 Barcelona Institute for Global Health (ISGlobal), Hospital Clínic, Universitat de Barcelona, Carrer Roselló 132, 08036 Barcelona, Spain; angela.martinez@isglobal.org (Á.M.-P.); ana.requena@isglobal.org (A.R.-M.); 9 Centre d’Atenció Primaria Casanova, Consorci d’Atenció Primària de Salut de l’Eixample (CAPSBE) Casanova, Carrer Rosselló 161, 08036 Barcelona, Spain; 10 Tropical Diseases, International Health and International Traveler Attention Unit, Clinic Hospital of Barcelona, 08036 Barcelona, Spain; ivera@clinic.cat; 11 Biomedical Research Networking Center (CIBER) of Infectious Diseases, Carlos III Health Institute (CIBERINFEC, ISCIII), Carrer Melchor Fernández Almagro, 3, 28029 Madrid, Spain; 12 Department of Medicine Solna, Karolinska Institutet, Solnavägen 1, 17177 Solna-Stockholm, Sweden; 13 Department of Infectious Diseases, Karolinska University Hospital, Solnavägen 1, 17177 Solna-Stockholm, Sweden; 14 Department of Genetics and Microbiology, Universitat Autònoma de Barcelona, 08208 Sabadell, Spain; 15 Department of Medicine, Universitat Autònoma de Barcelona, 08208 Sabadell, Spain

**Keywords:** international travel, extra-European countries, rectal colonization, extended-spectrum betalactamase, carbapenemase, methicillin-resistant *Staphylococcus aureus*

## Abstract

Multidrug-resistant (MDR) bacteria have become one of the most important health problems. We aimed to assess whether international travel may facilitate their spread through the colonization of asymptomatic travelers. A cross-sectional study was conducted (November 2018 to February 2022). Pharyngeal and rectal swabs were obtained from long-term travelers and recently arrived migrants from non-European countries, and an epidemiological survey was performed. Colonization by Gram-negative bacteria and methicillin-resistant *Staphylococcus aureus* (MRSA) was determined by chromogenic media and MALDI-TOF-MS. Resistance mechanisms were determined by the biochip-based molecular biology technique. Risk factors for colonization were assessed by logistic regression. In total, 122 participants were included: 59 (48.4%) recently arrived migrants and 63 (51.6%) long-term travelers. After their trip, 14 (11.5%) participants—5 (8.5%) migrants and 9 (14.3%) travelers—had rectal colonization by one MDR bacterium. *Escherichia coli* carrying the extended-spectrum beta-lactamase (ESBL) CTX-M-15 was the most frequent. No participants were colonized by MRSA or carbapenemase-producing Enterobacteriaceae. The only risk factor independently associated with MDR bacterial colonization was previous hospital attention [OR, 95% CI: 10.16 (2.06–50.06)]. The risk of colonization by MDR bacteria among recently arrived migrants and long-term travelers is similar in both groups and independently associated with previous hospital attention.

## 1. Introduction

The struggle against antibiotic resistance has become a global priority. According to the World Health Organization (WHO) [1], some of the most worrisome microorganisms are carbapenem-resistant *Acinetobacter*, *Candida auris*, *Clostridioides difficile*, carbapenem-resistant Enterobacteriaceae (CRE), extended-spectrum beta-lactamase-producing *Enterobacteriaceae* (ESBL-E), vancomycin-resistant Enterococci (VRE), multidrug-resistant *Pseudomonas aeruginosa*, methicillin-resistant *Staphylococcus aureus* (MRSA), and drug-resistant *Streptococcus pneumoniae*. Multidrug-resistant (MDR) bacteria spread is enabled by different types of interactions between colonized and non-colonized hosts, especially in certain settings such as the healthcare system [2]. In both the hospital and community settings, antibiotic use has been identified as one of the most important risk factors for colonization by multidrug-resistant microorganisms [3]. 

MDR bacterial colonization is a known risk factor for subsequent infections [4]. In some scenarios like MRSA carriage, decolonization is considered an effective strategy for reducing the risk of systemic infections [5]. 

Infections due to MDR bacteria are associated with increased mortality, an extended length of hospitalization, and higher costs [6]. They cause as many as 1.27 million deaths per year [7], in part because of the lack of appropriate antibiotics to combat them [8]. Delays in initiating appropriate empirical treatment have been associated with worse prognoses in patients affected by severe infections caused by MDR bacteria [9]. As MDR bacterial infections are frequently preceded by colonization, improving the identification of patients at risk is crucial [10]. Thus, after the detection of MDR bacterial colonization, measures to reduce spread can be applied [11]. This information may also be of interest for minimizing empirical treatment errors. 

In the community setting, factors associated with consumable water, agriculture, livestock, veterinarian health, the environment, or diet have also been strongly associated with MDR bacterial transmission [12]. The concept of One-Health was established as a way of stressing the importance of all these factors interacting simultaneously [13]. 

International travel to regions with a high burden of MDR bacteria has also been identified as a risk factor for MDR bacterial colonization [14]. The Western Pacific, Eastern Mediterranean, and Southeast Asian regions show the highest MDR bacterial colonization rates worldwide, while Africa and Central and South America have the lowest [15]. Diarrhea and antibiotic consumption while traveling are some of the factors usually associated with colonization by MDR bacteria [16,17]. However, the travel characteristics specifically associated with an increased risk of colonization are poorly defined to date. 

The aim of this study was to analyze the prevalence of colonization by ESBL-E, CRE, or MRSA in long-term travelers and migrants who had recently arrived in our country in order to establish the risk factors for colonization and compare the two cohorts.

## 2. Materials and Methods

### 2.1. Study Design and Participants

We conducted this multicenter, cross-sectional study in adults (>18 years) who met one of the following conditions: (i) long-term travelers who planned a stay of three months or longer in a non-European country or who had returned in the previous three months, and (ii) recently arrived migrants who had arrived in Europe from a non-European country also in the previous three months. Participants were seen at one of the five outpatient clinics involved in this study (the tropical diseases services of Hospital Parc Taulí and the Hospital Clínic of Barcelona, and three primary care centers: El Clot, Adrià 5A Marc Aureli, and Casanova), all of them located in the region of Barcelona, from June 2018 to May 2022.

### 2.2. Procedures

All participants who met the inclusion criteria and provided signed informed consent filled in a questionnaire with personal and socio-demographic information as well as the characteristics of their stays outside Europe. Among travelers, those who were seen before their travel outside Europe were scheduled for a follow-up appointment immediately upon their return.

Oropharyngeal and rectal swabs (Copan eSwab, Copan, Brescia, Italy) were obtained from all participants after their stays abroad or on arrival. Long-term travelers seen before their journeys also provided samples prior to their departure. 

### 2.3. Microbiological Procedures

The rectal samples were directly inoculated onto the selective chromogenic media ChromID ESBL^®^ and ChromID CARBA SMART^®^ (bioMérieux, Marcy l’Etoile, France) to detect MDR bacteria defined as Gram-negative bacteria resistant to third-generation cephalosporins or carbapenems.

The oropharyngeal samples were inoculated onto the selective chromogenic medium chromID_MRSA^®^ (bioMérieux, Marcy l’Etoile, France) to detect MRSA. Bacterial growth within a 48 h period was identified with MALDI-TOF MS (MALDI-TOF MS (Bruker Daltonics, Bremen, Germany). The in vitro antimicrobial susceptibility testing method was performed using MicroScan Walkaway^®^ (Beckman Coulter, Brea, CA, USA), and the interpretation of the antimicrobial susceptibility testing results was performed according to EUCAST criteria [18]. DNA extraction was performed using the DNeasy Blood & Tissue Purification Kit (QIAGEN GmbH, Hilden, Germany), and the resistance mechanisms of the isolated Enterobacteriaceae were determined using a microarray (Check-Points BV, Wageningen, Netherlands) assay that was used for the simultaneous detection of genes encoding clinically important carbapenemases and ESBLs. This assay was performed according to the manufacturer’s recommendations (Check-Points BV, Wageningen, The Netherlands). Briefly, the extracted DNA was ligated with probes that recognized specific resistance genes and contained unique tags. These probes (including the unique tags) were amplified in a multiplex PCR. The resulting products were detected with a tube microarray, in which the unique tags localized the amplification products to specific locations on the microarray. 

Check-MDR CT103XL^®^ is a qualitative in vitro diagnostic test based on detecting multiple genetic variations encoding different resistance mechanisms through specific probe hybridization, the subsequent amplification of these products, and visualization through colorimetric detection. This diagnostic system relies on the specific molecular recognition of a target DNA and its subsequent amplification using universal primers. Each DNA sequence is recognized by a probe containing a ZIP code corresponding to a specific position within the array, allowing the presence or absence of the genes of interest to be detected. Those that are bound will be amplified and, subsequently, hybridized on the array. Visualization is achieved through colorimetric detection, generating an image that is analyzed using software to obtain an objective and definitive assay result [19]. Specifically, genes for the IMP, KPC, VIM, NDM, and SME carbapenemases as well as the OXA-48-like, CTX-M group, SHV and AmpC, β-lactamases were screened.

### 2.4. Statistical Analyses

Continuous variables were reported as medians and interquartile ranges (IQRs) and compared using Student’s *t*-test or the Mann–Whitney U-test, as appropriate. Categorical data were reported as numbers and percentages and compared using the chi-squared test. Survival analysis was performed by Kaplan–Meier analysis. The risk factors for colonization by multidrug-resistant microorganisms were analyzed by using a logistic regression model with comparisons reported with odds ratios (ORs) at 95% confidence intervals (CIs). For all tests, a *p*-value of <0.05 was considered significant. The statistical analyses were performed using Stata statistical package v.14 (Stata Corporation LLC).

### 2.5. Ethics

All participants provided signed informed consent. This study was approved by the Medical Research Ethics Committee at the Institut d’Investigació I Innovació Parc Taulí, Sabadell (ref. 2017/653).

## 3. Results

### 3.1. Participant Characteristics

In total, 122 participants were included, 81 (66.3%) of them being women. The median age was 29 years (IQR: 25–36), and the median height and weight were 167 cm (IQR: 160–175) and 67.4 kg (IQR: 55–77), respectively. A total of 26 (21.3%) participants had chronic diseases: hypertension (6), diabetes (2), human immunodeficiency virus infection (2), inflammatory bowel disease (1), chronic lung disease (2), chronic liver disease (1), hypothyroidism (5), hiatal hernia (1), dyslipidemia (1), multiple sclerosis (1), depression (1), obesity (1), migraine (1), epilepsy (1), folic acid deficiency (1), cervical pain (1), sprue (1), systemic lupus erythematosus (1), and obesity (1).

Regarding toxic habits, 22 (18%) smoked cigarettes, 54 (46.5%) drank alcohol, and 12 (10.3%) consumed other types of drugs (9 consumed cannabis, 2 recreational drugs, and 1 hallucinogenic mushrooms). During the previous year, 14 (11.5%) participants had required hospital admission or emergency attention, and 2 (1.6%) had undergone surgery. Also, 13 (10.7%) participants had taken probiotics, and 39 (32%) had been prescribed antibiotics. Two participants were taking antibiotics at inclusion. 

Dietary habits, defined as the types of food usually consumed three or more days per week, were recorded as follows: 81 (66.4%) consumed vegetables, 76 (62.3%) fruit, 59 (74.2%) meat, 71 (58.2%) eggs, 67 (54.9%) dairy products, 54 (44.3%) legumes, 37 (30.3%) fish, and 25 (20.5%) saturated fats, and 2 (1.6%) participants were vegetarian or vegan. 

### 3.2. Travel Characteristics

In total, 63 (51.6%) long-term travelers and 59 (48.3%) recently arrived migrants arrived from non-European countries. 

Among the travelers, 43 (68.3%) arrived from America, 14 (22.2%) from Asia, and 6 (9.5%) from Africa. Their most frequent reason for travel was tourism (14 (38.8%)), followed by visiting family (8 (22.2%)), cooperation (7 (19.4%)), and study (7 (19.4%)). Foreign stays were predominately in urban areas (11 (25.6%)), while visits to rural areas (8 (18.6%)) were less frequent. The majority of travelers (24 (58.7%)) visited both destinations. During their stays abroad, 26 (28.2%) participants were guests in tourist accommodations, while 7 (21.2%) stayed in private houses. In total, 32 (50.8%) were in close contact with the autochthonous population, and 37 (58.7%) had some contact with animals.

Among the recently arrived migrants, 34 (60.7%) arrived from America, 12 (21.4%) from Asian countries, and 10 (17.9%) from Africa. In total, 51 (91.1%) lived in urban areas and 4 (7.1%) in rural areas. All of them were hosted in households, and 35 (60.3%) had contact with animals. 

According to the Bristol stool scale, after their journeys, 78 participants (82.1%) had formed stools, while 17 (17.8%) had unformed stools or diarrhea. 

### 3.3. MDR Bacterial Colonization

In total, 14 (11.2%) participants had rectal colonization by one MRD bacterium after their arrival (Table 1). Their characteristics are summarized in Table 1. The microorganisms isolated were ESBL-producing *E. coli* (10), Pseudomonas aeruginosa (2), ESBL-producing *Klebsiella pneumoniae* (1), and *Citrobacter* spp. (1). None of the participants had oropharyngeal colonization by MRSA or rectal colonization by carbapenemase-producing *Enterobacteriaceae*.

The beta-lactamase types among ESBL-producing *E. coli* were CTX-M-15 (6), CTX-M1 (2), CTX-M-9 (1), TEM (1) and TEM WT (1), and SHV 238S + 240K (1), while the only *K. pneumoniae* isolate produced was SHV-238+.

Rectal samples were obtained from 35 travelers before their departure. One of them (2.9%) was colonized with ESBL-producing *E. coli* (CTX-M-9), but none of them were after returning to Europe. The obtained relative risk for traveling was 6.15 (IC 95%: 0.91–41.54).

### 3.4. Risk Factors for MDR Bacterial Colonization (Table 2)

Among the participants colonized by MDR bacteria, 5 (35.5%) were recently arrived migrants, and 9 (64.3%) were travelers. There were no differences between the groups (*p* = 0.319). Regarding the origin of travel, 8 came from America (1 from Colombia, 1 from Peru, 2 from Brazil, 2 from Bolivia, 1 from Paraguay, and 1 from the United States) (the colonization rate was 10%), 5 from Asia (2 from Nepal, 1 from India, 1 from Indonesia, and 1 from Taiwan) (the colonization rate was 19%), and 1 from Africa (Nigeria) (the colonization rate was 6%). One of the three participants being treated with proton-pump inhibitors and four of the fourteen participants who had received antibiotics in the previous year were colonized by MDR *Enterobacteriaceae*.

The univariate analysis identified the following risk factors for colonization: coming from a rural area (*p* = 0.039), hospital attention during the last year (*p* = 0.008), and chronic disease (*p* = 0.039). Differences between origin countries or types of participants were not found. The multivariate analysis identified hospital attention during the last year as the only independent risk factor.

**Table 2:** Risk factors for colonization by multidrug-resistant microorganisms.

Variables	Categories	Total	Non-Colonized	Colonized	*p*	Multivariate OR (IQR 95%)
Participant type	Recently arrived migrants	59	54 (91.5)	5 (8.5)		
	Long-term traveler	63	54 (85.7)	9 (14.3)	0.319	
Sex	Male	39	34 (87.2)	5 (12.8)		
	Female	81	72 (88.9)	9 (11.1)	0.314	0.62 (0.13–3.05)
Age (Md, IQR)		29 (25–36)	29 (25–36)	29 (25.5–34.5)	0.920	0.98 (0.91–1.05)
Height (Md, IQR)		167 (160–175)	167 (160–178)	166 (155–174)	0.497	
Weight (Md, IQR)		67.49 (55–77)	57 (55.5–77.5)	67 (51–72.5)	0.614	
Education level	Pre-university	6	5 (83.3)	1 (16.7)		
	University	60	54 (90)	6 (10)	0.617	
Profession	Rural job/veterinarian	8	6 (75)	2 (25)		
	Urban job	22	20 (86.4)	2 (13.6)	0.275	
	Student	36	33 (91.7)	3 (8.3)	0.201	
E. Bristol Type	Unformed feces or diarrhea	17	15 (88.2)	2 (11.8)		
	Solid	78	70 (89.7)	8 (10.3)	0.854	
Destination/origin of travel	Asia	26	21 (80.8)	5 (19.2)		
	Africa	16	15 (93.8)	1 (6.3)	0.267	
	America	77	68 (89.6)	9 (10.4)	0.247	
Hospital attention in the last year	No	103	94 (91.3)	9 (8.7)		
	Yes	14	9 (64.3)	5 (35.7)	0.008	10.16 (2.06–50.06)
Chronic diseases	No	89	82 (92.1)	7 (7.9)		
	Yes	26	20 (76.9)	6 (23.1)	0.039	2.78 (0.53–14.51)
Antibiotic intake during the last year	No	78	68 (87.2)	10 (12.8)		
	Yes	39	35 (89.7)	4 (10.3)	0.688	
Probiotic intake during the last year	No	103	91 (87.5)	13 (12.5)		
	Yes	13	12 (92.3)	1 (7.7)	0.618	
Accommodation type	Tourist accommodation	26	21 (80.8)	5 (19.2)		
	Household	66	61(92.4)	5 (7.6)	0.319	
Area visited/area of origin	Rural	12	8 (66.7)	4 (33.3)		
	Urban	62	56 (90.3)	6 (9.7)	0.039	0.21 (0.04–1.22)
	Both	25	23 (92)	2 (8)	0.068	
Animal contact	No	49	44 (89.8)	5 (10.2)		
	Yes	72	63 (87.5)	9 (12.5)	0.699	
Close contact with native population	No	31	27 (87.1)	4 (12.9)		
	Yes	91	81 (89)	10 (11)	0.773	

## 4. Discussion

This study is a comprehensive description of the prevalence and risk factors for MDR bacterial colonization among long-term travelers and recently arrived migrants. The incidence of nasal colonization by MRSA was negligible, while that of rectal colonization by MDR Enterobacteriaceae was close to 11% and associated with previous hospitalization, but not with recent antibiotic intake, travel diarrhea, or the country of provenance. No differences were observed between long-term travelers and recently arrived migrants. 

In a recent meta-analysis that assessed the risk factors for multidrug-resistant Enterobacteriaceae colonization during international travel [20], this colonization was closely related to the region visited, with the highest rates being recorded in Asia: 71% in southern Asia, and 36% in the rest of the continent. The proportion of colonized participants who had traveled from Central and South America was significantly lower, with an incidence of 12%, similar to our study, in which American countries were the most frequent destinations for travelers and recently arrived migrants. 

The duration of travel may also be an important factor for colonization. A recent study showed that the number of travelers with temporary colonization during their journeys exceeded that of travelers still colonized after return. The mean time needed for ESBL-E colonization was eight days (95% CI: 5–10) [21]. These results suggest that colonization rates may follow a downward trend during travel. However, in a study of 192 Japanese business travelers who stayed predominantly in Asia and Africa for six months or longer, Mizuno et al. [22] reported a colonization rate after return close to 40%, quite similar to the rates reported in studies of shorter-term travelers and higher than the ones that we observed.

Our finding of a low level of carbapenemase-producing Enterobacteriaceae colonization is in agreement with the results of most other studies on the issue [16]. When detected, it was found in participants who had traveled to Asia [21,23,24]. MRSA colorization was assessed in 58 US travelers to Mexico, Central America, and the Caribbean [25,26], with a median length of 12 days, and, as in our study, none of the subjects were colonized with MRSA. As for ESBL genes, our study and previous work [15,27] found the most frequent to be CTX-M-15, followed by CTX-M-9. 

The risk factors for different types of MDR bacterial colonization among travelers have been previously assessed. Among them, diarrhea [16,20,24,28] and antibiotic intake by travelers during travel [14,16,21] have been related to increased incidence. However, similar to us, other authors have found no associations with any of these predictors [29]. Interestingly, we did observe an association with travel to a rural area, for which we have no plausible explanation. We might hypothesize an accumulation of risk factors among the colonized patients in this cohort, as they were frequently long-term travelers who had contact with animals and, in most cases, had received hospital attention in the previous year. Also, in our study, some of the classically described risk factors [30], such as previous hospitalization or chronic diseases, were related to colonization with MDR Gram-negative bacteria. Importantly, the former was the unique independent risk factor identified. Unfortunately, we do not have a second sample to assess whether the colonization rate decreased or not. However, a recent study showed a progressive reduction in MDR bacterial colonization after hospital admission, reaching around 39% after one year [31].

We stress that we did not find any differences between recently arrived migrants and long-term travelers regarding colonization rates. However, both cohorts had an increased incidence of colonization compared with participants who had not yet traveled. This is an important finding, as it reinforces the message that these two populations may not need different screening strategies per se.

Our study has some limitations. First, a low sample size was achieved, and they constituted a heterogeneous sample in terms of their origins, destinations, and reasons for travel. Among travelers, a low proportion had samples collected before traveling, making it challenging to establish a baseline colonization status. Post-travel samples were collected within the first month but not immediately upon arrival, which may increase the likelihood of colonization within our country. Also, the rectal samples were self-obtained without supervision. Although detailed instructions were given, we cannot be sure that all of them were collected as required. Last, although MDR bacterial flora can change over time, we did not conduct further assessments of colonization beyond the first month after arrival. In spite of these limitations, our study contributes to the understanding of MDR bacterial colonization in non-European long-term travelers and foreigners, showing lower proportions in Asia and Africa than in studies performed elsewhere. Our research also contributes to the study of a little-examined population, travelers from America, finding a similar rate to the European population. Finally, our results support that if screening interventions for MDR bacterial colonization are necessary, they should be similarly implemented in both foreigners and long-term travelers. However, cost-effectiveness studies are needed to identify individuals for which targeted screening is the most suitable and appropriate strategy.

## Figures and Tables

**Table 1:** Demographic and travel characteristics of long-term travelers and recently arrived migrants colonized by multidrug-resistant bacteria.

	MDR Bacterial Colonization	ESBL Gene	Patients’ Characteristics							Travel Characteristics		
			Age/Sex	Comorbidities	Toxic Habits	Hospital Attention (Last Year)	Antibiotics (Last Year)	Surgeries (Last Year)	Contact with Animals	Country	Type of Participant	Type of Destination
1	*E. coli*	CTX-M15	31/female	No	Alcohol	Yes	Yes	No	Yes	India	Long-term traveler	Urban and rural
2	*E. coli*	CTX-M15	30/female	No	No	No	No	No	Yes	Nepal	Long-term traveler	Urban
3	*E. coli*	CTX-M1	30/female	No	Alcohol (wine)	Yes	Yes	No	Yes	Belize, Costa Rica, El Salvador, Guatemala, Honduras, Mexico, and Nicaragua	Long-term traveler	Urban and rural
4	*E. coli*	TEM and CTX-M1	23/female	No	Alcohol and tobacco	Yes	Yes	No	Yes	Nepal	Long-term traveler	Rural
5	*E. coli*	SHV 238S + 240K and TEM WT	56/male	Yes (hypertension and dyslipidemia)	No	No	No	No	Yes	Peru	Recently arrived migrant	Rural
6	*E. coli*	CTX-M9	29/female	No	No	No	No	No	No	Taiwan	Recently arrived migrant	Urban
7	*K. pneumoniae*	SHV-238	30/female	NA	Alcohol	NA	No	No	No	Nigeria	Recently arrived migrant	Urban
8	*E. coli*	CTX-M15	24/male	No	Alcohol	Yes	Yes	No	Yes	USA	Recently arrived migrant	Urban
9	*E. coli*	CTX-M15	42/female	Yes (hypothyroidism)	No	No	No	No	No	Colombia	Recently arrived migrant	Urban
10	*E. coli*	CTX-M15	30/male	Yes (HIV)	Alcohol, cocaine, and other recreational drugs	No	No	No	Yes	Paraguay	Recently arrived migrant	Urban
11	*E. coli*	CTX-M15	32/male	Yes (epilepsy)	No	Yes	No	No	Yes	Brazil, Colombia, Bolivia, and Peru	Long-term traveler	Rural
12	*Pseudomonas* spp.	-	24/male	No	Alcohol and tobacco	No	No	No	Yes	Indonesia, Philippines, and Vietnam	Long-term traveler	Rural
13	*Pseudomonas* spp.	-	37/female	Yes (celiac disease)	No	No	No	No	NA	Bolivia	Long-term traveler	NA
14	*Citrobacter* spp.	-	38/female	Yes (obesity)	No	No	No	No	NA	Brazil	Long-term traveler	NA

MDR: multidrug-resistant bacteria, ESBL: *Escherichia coli* carrying extended-spectrum beta-lactamase, NA: not apported, and HIV: human immunodeficiency virus-infected.

## Data Availability

Data are contained within the article.
